# Trophic factors as modulators of motor neuron physiology and survival: implications for ALS therapy

**DOI:** 10.3389/fncel.2014.00061

**Published:** 2014-02-28

**Authors:** Luis B. Tovar-y-Romo, Uri Nimrod Ramírez-Jarquín, Rafael Lazo-Gómez, Ricardo Tapia

**Affiliations:** División de Neurociencias, Instituto de Fisiología Celular, Universidad Nacional Autónoma de MéxicoMexico City, Mexico

**Keywords:** amyotrophic lateral sclerosis, spinal cord neurodegeneration, motor neurons, neurotrophic factors, VEGF

## Abstract

Motor neuron physiology and development depend on a continuous and tightly regulated trophic support from a variety of cellular sources. Trophic factors guide the generation and positioning of motor neurons during every stage of the developmental process. As well, they are involved in axon guidance and synapse formation. Even in the adult spinal cord an uninterrupted trophic input is required to maintain neuronal functioning and protection from noxious stimuli. Among the trophic factors that have been demonstrated to participate in motor neuron physiology are vascular endothelial growth factor (VEGF), glial-derived neurotrophic factor (GDNF), ciliary neurotrophic factor (CNTF) and insulin-like growth factor 1 (IGF-1). Upon binding to membrane receptors expressed in motor neurons or neighboring glia, these trophic factors activate intracellular signaling pathways that promote cell survival and have protective action on motor neurons, in both *in vivo* and *in vitro* models of neuronal degeneration. For these reasons these factors have been considered a promising therapeutic method for amyotrophic lateral sclerosis (ALS) and other neurodegenerative diseases, although their efficacy in human clinical trials have not yet shown the expected protection. In this minireview we summarize experimental data on the role of these trophic factors in motor neuron function and survival, as well as their mechanisms of action. We also briefly discuss the potential therapeutic use of the trophic factors and why these therapies may have not been yet successful in the clinical use.

## Introduction

Neuronal development and survival depend on a balanced and tightly regulated support from trophic factors. Such factors are capable of regulating several important physiological processes, such as neuronal differentiation, maintenance of synapses, neuronal survival through the inhibition of apoptosis, neurogenesis and axonal outgrowth (Korsching, 1993; Boonman and Isacson, 1999; Hou et al., 2008). In addition, they provide an environmental niche suitable for neuronal survival (Mudò et al., 2009). Trophic support is essential for neurons in the spinal cord and is conferred from many different cellular sources including astrocytes, microglia, neurons and endothelial cells (Ikeda et al., 2001; Béchade et al., 2002; Dugas et al., 2008; Su et al., 2009; Hawryluk et al., 2012). Therefore, trophic support is considered a promising therapeutic strategy for neurodegenerative diseases (Kotzbauer and Holtzman, 2006), and it plays an important role in cellular therapy aimed at the reinnervation of lost neuromuscular synapses (Casella et al., 2010).

Amyotrophic lateral sclerosis (ALS) is caused by the selective and progressive loss of spinal, bulbar and cortical motor neurons that lead to irreversible paralysis, speech, swallowing and respiratory malfunctions and eventually death of the affected individuals in a rapid disease course. ALS is mostly sporadic with 90% of the cases occurring without a family history of the disease. However, in the recent years it has become evident that many sporadic cases carry alterations in proteins that have been found mutated in familial cases that might, at least, increase the probability for developing ALS (Deng et al., 2010). Many of these mutations involve alterations in the TAR DNA-binding protein 43 (TDP43) and Fused in sarcoma (FUS) genes that bind RNA molecules (Gordon, 2013; Sreedharan and Brown, 2013), whereas most familial cases with a dominant autosomal inheritance pattern are caused by mutations in superoxide dismutase 1 (SOD1; Rosen et al., 1993). Transgenic mice expressing a mutant form of the human SOD1 are the most widely used model for *in vivo* studies of ALS (Gurney et al., 1994). Trophic factors have been thought as therapeutic targets for ALS, aiming at restoring lost neuromuscular synapses and rescuing motor neurons from toxicity.

There is a series of well characterized trophic factors for the CNS, such as brain-derived neurotrophic factor (BDNF), insulin-like growth factor 1 (IGF-1), ciliary neurotrophic factor (CNTF), glial-derived neurotrophic factor (GDNF), nerve growth factor (NGF), growth hormone and vascular endothelial growth factor (VEGF). Many of these have been tested for neuroprotective potential in different experimental models of ALS. In fact, viral vectors encoding growth factors are among the most effective ways to delay the progression of degenerative processes and prolong survival in ALS mice (Wang et al., 2002; Kaspar et al., 2003; Azzouz et al., 2004; Dodge et al., 2008).

### Trophic factors during motor neuron development

Motor neuron development is differentially affected by specific trophic factor shortage, and loss of particular trophic signaling alters the development of different subpopulations of motor neurons in heterogeneous ways. The absence of GDNF alters the location of developing motor neurons that innervate the limbs in the spinal cord (Haase et al., 2002; Kramer et al., 2006) and selectively affects the innervation of intrafusal muscle spindles (Gould et al., 2008). Interestingly, the overexpression of this factor in muscle during development causes a hyperinnervation of neuromuscular junctions (Nguyen et al., 1998). In contrast, BDNF may not be as important for motor neurons, because although the lack of this trophic factor severely affects the normal development of sensory neurons, motor neurons are able to develop without major alterations (Ernfors et al., 1994a; Jones et al., 1994). Furthermore, distinct motor neuron subpopulations show different sensitivities to the lack of neurotrophins. For example, the absence of neurotrophin-3 produces a complete loss of spinal motor neurons while facial motor neurons are spared (Ernfors et al., 1994b; Gould et al., 2008), and the absence of CNTF produces no alterations for motor neuron development at the spinal or cranial levels (DeChiara et al., 1995), although the loss of its receptor CNTFRα generates severe motor neuron deficits and mice lacking this receptor die perinatally (DeChiara et al., 1995). A possible alternate ligand for this receptor is the dimer formed by cardiotrophin-like cytokine/cytokine-like factor 1, whose deletions have been shown to cause a significant reduction in the number of motor neurons (Forger et al., 2003). The absence of other factors such as cardiotrophin-1 has also been reported to produce a significant loss of motor neurons (Oppenheim et al., 2001; Forger et al., 2003), and the loss of IGF-1 causes significant reduction in the number of trigeminal and facial motor neurons (Vicario-Abejón et al., 2004). Finally, while the lack of VEGF is lethal, a deletion of the hypoxia response element in the promoter region of the VEGF gene causes a decrease in the expression of this factor that leads to an adult-onset progressive loss of motor neurons in mice (Oosthuyse et al., 2001). After this fortuitous discovery, it was reported that certain VEGF haploytpes (-2578C/A, -1154G/A and -634G/C) conferred an increased susceptibility to ALS in humans, but later on in a meta-analysis conducted with more than 7000 subjects from at least eight different populations no association between these haplotypes and ALS was found (Lambrechts et al., 2009). Moreover, no mutations in the hypoxia response element of the VEGF promoter (Gros-Louis et al., 2003), or in the VEGF receptor 2 (Brockington et al., 2007) were found in ALS patients.

Neurotrophic factors are not only important during development, but they also regulate motor neuron maintenance and survival even long after neurons have become fully differentiated. As well, they might be able to trigger the activation of endogenous regenerative processes. Aside from the synthesis of trophic factors in the local spinal microenvironment, synaptic targets of motor neurons also play important roles in the trophic feedback. As a matter of fact, this is an essential event for the development of the CNS during which originating neurons receive trophic input from their target tissues that enables them to surpass an endogenous-codified programmed cell death (Oppenheim, 1991). In the case of motor neurons these effects are mostly mediated by skeletal muscle-derived factors (Oppenheim et al., 1988; Grieshammer et al., 1998; Kablar and Rudnicki, 1999).

### Trophic factor effects on motor neuron survival

Among all the trophic factors tested in experimental ALS models, VEGF has been shown to be one of the most potent motor neuron protectors. VEGF remarkably retards the progression of the disease and the loss of motor neurons in familial (Azzouz et al., 2004; Zheng et al., 2004; Storkebaum et al., 2005; Wang et al., 2007), as well as in sporadic (Tovar-Y-Romo et al., 2007; Tovar-Y-Romo and Tapia, 2010, 2012) experimental models of motor neurodegeneration.

Activation of VEGF receptor 2 triggers the phosphorylation of intracellular pathways driven by phosphatidyl-inositol-3-kinase (PI3-K), phospholipase C-γ, and mitogen-activated protein kinase (MEK) that promote the inhibition of pro-apoptotic factors like Bad (Yu et al., 2005) and caspases 9 (Cardone et al., 1998) and 3 (Góra-Kupilas and Joško, 2005; Kilic et al., 2006). The activation of these intracellular signaling pathways has been extensively studied in the CNS (Zachary, 2005). VEGF-dependent activation of PI3-K/Akt is sufficient to prevent motor neuronal death in familial models of ALS *in vitro* (Li et al., 2003; Koh et al., 2005; Tolosa et al., 2008) and in experimental *in vitro* models of excitotoxic neuronal death (Matsuzaki et al., 2001). Furthermore, the activation of PI3-K/Akt is required for motor neuron survival and axonal regeneration after spinal cord injury (Namikawa et al., 2000). We have demonstrated that the signaling mediated by PI3-K is critically involved in the protective effect of VEGF against AMPA-induced excitotoxic spinal neurodegeneration *in vivo* (Tovar-Y-Romo and Tapia, 2010).

VEGF also mediates neuroprotection through the inhibition of stress activated protein kinases like p38 mitogen-activated protein kinase. Increased levels of phosphorylated p38 have been found in motor neurons and glia in the familial mouse model of ALS (Tortarolo et al., 2003; Holasek et al., 2005; Veglianese et al., 2006; Dewil et al., 2007), even at the pre-symptomatic stage (Tortarolo et al., 2003), and p38 is also an important factor in a cell death pathway specific for motor neurons (Raoul et al., 2006). Interestingly, the inhibition of p38 prevents motor neuron death in an *in vitro* familial model of ALS (Dewil et al., 2007), and we and others have proven that VEGF can suppress p38 activation in both familial (Tolosa et al., 2009) and excitotoxic (Tovar-Y-Romo and Tapia, 2010) models of spinal cord neurodegeneration.

An increased expression of the VEGF-inducing factor Hypoxia induced factor 1 (HIF-1α) in the spinal cord may occur due to relative hypoxic conditions that exist in the spinal microenvironment, although motor neurons seem to be unable to fully respond to increased downstream effectors such as VEGF (Sato et al., 2012). One possible explanation for this and for the decrease of VEGF levels found in human patients (Devos et al., 2004) might be that inducing factors such as HIF-1α are prevented from translocating to the nucleus even though their concentrations are increased in the cytoplasm (Nagara et al., 2013). This failure to mount the complete response of VEGF synthesis during hypoxia is not cell type specific and it has been demonstrated to occur in monocytes from ALS patients (Moreau et al., 2011).

In contrast to the good protection potential of VEGF, other factors like BDNF failed to protect in different experimental paradigms. BDNF is synthesized by activated microglia in the first stages of the disease when the glial response mainly exerts anti-inflammatory and protective effects, but its production is lost when microglia turns toxic at later stages (Liao et al., 2012). In addition, BDNF does not protect motor neurons from excitotoxicity in experimental models *in vitro* (Fryer et al., 2000) and *in vivo* (Tovar-Y-Romo and Tapia, 2012). This could be possibly due to the sequestration of the ligand by a truncated isoform of the high affinity receptor that is known to be expressed in motor neurons, because removing this truncated receptor significantly delays the disease onset in the mouse familial model (Yanpallewar et al., 2012). In spite of this, BDNF may be a risk factor for neurons by increasing their sensitivity to excitotoxicity (Fryer et al., 2000), or through the activation of NADPH oxidase (Kim et al., 2002), an enzyme involved in motor neuron pathology by damaging the survival pathways activated by trophic factors (Wu et al., 2006). Other growth factors have also been shown to be beneficial although to a lesser extent.

The expression of GDNF by astrocytes is up-regulated after spinal cord ischemia and this might be a mechanism of protection for motor neurons against excitotoxic death (Tokumine et al., 2003). GDNF exerts its neuroprotective effects preferentially on neuronal somas rather than on nerve endings at the neuromuscular synapse when it is administered directly in the spinal cord (Suzuki et al., 2007). Conversely, when it is administered directly in the muscle, GDNF preserves the muscle-nerve synapse and promotes motor neuron function and survival in a familial model of ALS (Suzuki et al., 2008), implying that the protective effects exerted by GDNF are rather limited by the proximity to the trophic source. Nonetheless, GDNF can be retrogradely transported along motor neuronal axons (Leitner et al., 1999), which allows the opportunity to explore a delivery route that will impact both somas and nerve endings. Interestingly, human ALS patients show an up-regulation of GDNF in muscle (Grundström et al., 1999), and the overexpression of GDNF in muscle but not in astrocytes extends lifespan in ALS mice (Mohajeri et al., 1999). Combined growth factor therapy might be an alternative that is worth exploring, as suggested by a recent report in the rat transgenic ALS model showing that VEGF and GDNF administered through an implant of human mesenchymal stem cells exert a synergistic protection in preserving nerve muscular synapses (Krakora et al., 2013).

In the case of CNTF, although the blockade of its expression has been reported to result in the loss of motor neurons and the development of motor symptoms (Masu et al., 1993), these effects are relatively mild when compared to those induced by the loss of other factors like VEGF. Interestingly, ALS patients have a selective decrease of CNTF expression in the CNS regions affected by the disease (Anand et al., 1995). Conversely, serum levels of CNTF are generally elevated in ALS patients, especially among those with the lumbar-onset form of the disease (Laaksovirta et al., 2008).

### Trophic factors as therapy for amyotrophic lateral sclerosis (ALS)

Clinical trials administering trophic factors to ALS patients have not been successful yet. Subcutaneous injections of CNTF, which was effective in the mutant mice models of motor neuron disease *pmn/pmn* (Sendtner et al., 1992) and wobbler (Mitsumoto et al., 1994), did not affect the progression of disease in humans, but caused minor adverse side effects (ALS CNTF Treatment Study Group, 1996). Similarly, disease progression was not modified in ALS patients treated with subcutaneous administration of BDNF (The BDNF Study Group, 1999). Two randomized double-blind placebo-controlled clinical trials administering recombinant human IGF showed little (Lai et al., 1997) or no effect (Borasio et al., 1998) on disease progression, even when IGF-1 was found to be protective in the transgenic rodent model of ALS (Kaspar et al., 2003; Dodge et al., 2008). A combined meta-analysis of both trials showed slight retardation in the disease progression in the group treated with IGF-1, although the results are not conclusive (Beauverd et al., 2012). Interestingly, it has been recently reported that skeletal muscle fiber production of IGF-1 is impaired in ALS patients (Lunetta et al., 2012), so that the modest effects found in some of the patients enrolled in the clinical trials might have been due to a compensation of impaired IGF-1 production by the exogenous administration of the factor. Finally, even when according to one report (Morselli et al., 2006) the majority of ALS patients showed deficiencies in growth hormone secretion, in a recent clinical trial the administration of this hormone to ALS patients did not produce any benefit as compared to patients that received placebo (Saccà et al., 2012).

The time of administration after symptom onset in a trophic factor-based therapy is critical. Trophic factors have a short time frame for protection of motor neurons once the noxious process is triggered and this is probably due to the rate at which motor neurons die during the time course of the disease. Histological studies of human spinal cord showed a large variability between the degree of motor neuron loss and muscle weakness (Stephens et al., 2006), and transgenic familial amyotrophic lateral sclerosis (FALS) mice bearing human (Dal Canto and Gurney, 1995; Bruijn et al., 1997) or murine (Morrison et al., 1998) mutant SOD1 do not present a significant loss of motor neurons prior to the onset of symptoms, and the neuronal loss occurs at a very fast rate over a period of 10 days. In our model of chronic spinal cord excitotoxicity we found that the onset of motor deficits, characterized by limping of the rear limbs, occurs before the loss of motor neurons, suggesting that the time at which the cellular death process starts but prior to clear neuronal degeneration constitutes a therapeutic frame within which growth factor administration could result effective (Tovar-Y-Romo et al., 2007; Tovar-Y-Romo and Tapia, 2012). In fact, in the FALS murine models the administration of VEGF (Azzouz et al., 2004; Storkebaum et al., 2005) or IGF-1 (Kaspar et al., 2003; Dodge et al., 2008) well before the beginning of symptoms confers a significantly better protection, observed by a delay in the progression of symptoms and increased lifespan, as compared to that produced when administered at the symptoms onset. A similar result was obtained in rats subjected to spinal AMPA-induced excitotoxicity, in which a delayed administration of VEGF clearly protected but only when administered before the beginning of motor deficit symptoms (Tovar-Y-Romo and Tapia, 2012). This difference possibly means that growth factors are helpful at preventing the accumulating toxicity that arises from neurodegenerative processes that begin before motor neuron death or symptoms onset (Dal Canto and Gurney, 1995; Bendotti et al., 2001). Unfortunately, obtaining a correct diagnosis of ALS is a complicated and slow process due to the many parameters needed to meet diagnosis criteria (Shook and Pioro, 2009; Bedlack, 2010), so that the earliest intervention with trophic factors once a patient is diagnosed may be already too late.

Administration routes for trophic factor therapy are also important. This is of special interest when considering that in the actual human disease cellular alterations take place along the entire spinal cord, which might be a target particularly difficult to reach. Therefore, assessing different ways to deliver trophic factors is worth trying. Intracerebroventricular (ICV) administration of VEGF has been proven efficient in the rat transgenic model of FALS (Storkebaum et al., 2005) and in our acute model of spinal cord excitotoxicity (Tovar-Y-Romo and Tapia, 2012). ICV administration has the capability to cover the entire spinal cord although it most probably creates a concentration gradient (Storkebaum et al., 2005). The continuous perfusion of trophic factors in the spinal cord by intrathecal infusions or into the brain by ICV injections overcome the blockade that the blood brain barrier represents for the delivery of these molecules. In fact, intrathecal injections have been tried in ALS patients for the delivery of IGF-1, with modest results (Nagano et al., 2005). Clinical trials for VEGF are now underway to assess the safety and tolerability of VEGF (Siciliano et al., 2010).

Other important aspects to consider in growth factor therapies are the stability of the molecule, the half-life of the proteins, the need for sustained delivery and exposure, the dose, their ability to cross the blood brain barrier, and the unwanted side effects on non-targeted cells (Suzuki and Svendsen, 2008). Nonetheless, the neuroprotective potential that growth factor represent overweighs the obstacles that need to be overcome in order to achieve a successful therapy.

## Conclusions

Because trophic support is an essential component for neuronal maintenance and survival, supplying motor neurons subjected to stressful or noxious stimuli with molecular factors that help them counteract cellular death processes, growth factors represent a therapeutic tool that is undoubtedly worth exploring for ALS. However, we still need to understand a great deal of the molecular pathways that cause growth factor shortage during the course of disease and the cellular and molecular mechanisms that limit the responses elicited by these factors when they are supplied exogenously. As well, we still need to identify proper therapeutic regimens and treatment approaches to be able to translate the findings we have made in experimental models into useful therapeutic procedures.

## Conflict of interest statement

The authors declare that the research was conducted in the absence of any commercial or financial relationships that could be construed as a potential conflict of interest.
